# Transplantation of free tibial periosteal grafts for the repair of articular cartilage defect: An experimental study

**DOI:** 10.4103/0019-5413.55973

**Published:** 2009

**Authors:** Ravijot Singh, Vijendra Chauhan, Neena Chauhan, Sansar Sharma

**Affiliations:** Department of Orthopaedic Surgery, Himalayan Institute of Medical Sciences, Swami Ram Nagar, Jollygrant, Doiwala, Dehradun, Uttarakhand - 248 140, India; 1 Department of Pathology, Himalayan Institute of Medical Sciences, Swami Ram Nagar, Jollygrant, Doiwala, Dehradun, Uttarakhand - 248 140, India

**Keywords:** Articular cartilage defects, periosteal grafts, transplantation

## Abstract

**Background:**

Articular chondrocytes have got a long lifespan but rarely divides after maturity. Thus, an articular cartilage has a limited capacity for repair. Periosteal grafts have chondrogenic potential and have been used to repair defects in the articular cartilage. The purpose of the present study is to investigate the differentiation of free periosteal grafts in the patellofemoral joint where the cambium layer faces the subchondral bone and to investigate the applicability of periosteal grafts in the reconstruction of articular surfaces.

**Materials and Methods:**

The study was carried out over a period of 1 year on 25 adult, male Indian rabbits after obtaining permission from the institutional animal ethical committee. A full-thickness osteochondral defect was created by shaving off the whole articular cartilage of the patella of the left knee. The defect thus created was grafted with free periosteal graft. The patella of the right knee was taken as a control where no grafting was done after shaving off the articular cartilage. The first animal was used to study the normal histology of the patellar articular cartilage and periosteum obtained from the medial surface of tibial condyle. Rest 24 animals were subjected to patellectomy, 4 each at serial intervals of 2, 4, 8, 16, 32 and 48 weeks and the patellar articular surfaces were examined macroscopically and histologically.

**Results:**

The grafts got adherent to the underlying patellar articular surface at the end of 4 weeks. Microscopically, graft incorporation could be appreciated at 4 weeks. Mesenchymal cells of the cambium layer were seen differentiating into chondrocytes by the end of 4 weeks in four grafts (100%) and they were arranged in a haphazard manner. Till the end of 8 weeks, the cellular arrangement was mostly wooly. At 16 weeks, one graft (25%) had wooly arrangement of chondrocytes and three grafts (75%) had columnar formation of cells. Same percentage was maintained at 32 weeks. Four grafts (100%) at 48 weeks showed columnar orientation. The control side showed no changes over the shaved off articular surface in all the rabbits. One rabbit at 4 weeks had a dislocation of the patella on the control side. None of the rabbits developed any infection or wound dehiscence.

**Conclusion:**

Autologous periosteal graft transplantation can be a promising substitute for articular cartilaginous defects.

## INTRODUCTION

The repair of defects in the articular cartilage such as those occurring in osteochondritis dessicans, depressed fracture of the tibial plateau, osteoarthrosis, and other injuries of the articular surface continue to present a difficult problem in joint reconstruction.

Clinical success of articular prostheses is overshadowed by their poor long-term performance, prohibitive cost, and technical difficulties posed by their use. It is important therefore to further research the possibilities of stimulating the production of an articular cartilage, especially in young patients in whom total arthroplasty is not a definitive solution.

It is well documented that the damaged articular cartilage has a very limited potential for healing, and articular defects larger than 2–4 mm in diameter rarely heal.^1^,^2^

Meachim and Roberts^3^ and Salter *et al*.^4^ observed that small deep defects extending into the subchondral bone could heal spontaneously through the ingrowth of the cartilaginous tissue from the subchondral bone. It has also been reported that free periosteal grafts may stimulate an enchondral bone formation^5^,^6^ and in a chondrotropic environment, favor cartilage formation.^6^ Furthermore, Rubak *et al*.^7^–^9^ demonstrated that free periosteal grafts transplanted to experimental defects in the rabbit femoral articular cartilage differentiated into hyaline-like cartilage of a histologically normal appearance.

The purpose of the present study is to investigate the differentiation of free periosteal grafts in the patellofemoral joint where the cambium layer faces the subchondral bone and to investigate the applicability of periosteal grafts in the reconstruction of articular surfaces.

## MATERIALS AND METHODS

A total of 25 adult (2.5–3.0 kg), male Indian rabbits were operated upon after obtaining permission from the institutional animal ethical committee.

The first rabbit was used to study the normal histology of the articular cartilage of the patella and periosteum from the medial surface of the tibial condyle. The remaining 24 were included in the experimental study. We selected the patellofemoral joint because of the following reasons: (1) the patello femoral joint is very unique. It is not a weight-bearing joint but nevertheless most complex and mechanically affected by the quadriceps muscle and the femoropatellar angle. Its normal function is easily disturbed as reflected by the high rates of chondromalacia and osteoarthritis.^10^ (2) It was easy for us to stitch the periosteum with the adjacent soft tissues to retain it *in situ*.

### Preoperative preparation

The rabbits were kept fasting for 3–4 h prior to surgery and then anesthetized using intramuscular ketamine (40 mg/kg) and diazepam (0.1–0.2 ml/kg). The average anesthesia and surgery time was about 30 min. Thirty minutes prior to surgery, each rabbit was given a bolus dose of cefotaxime injection (20 mg/kg body weight).

### Operative procedure

Under aseptic conditions, both the lower limbs were shaved and cleaned using 5% povidine–iodine solution and were draped using sterile towels.

#### Experimental side

**Figure 1:**
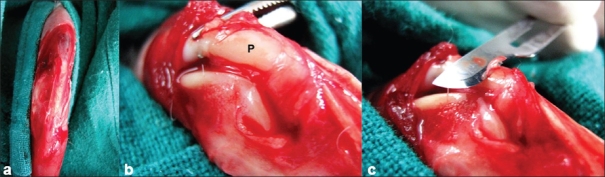
Clinical intraoperative photograph showing (a) medial and lateral parapatellar incisions; (b) the articular surface of the patella marked as “P”; (c) the articular surface of the patella being shaved off

**Figure 2:**
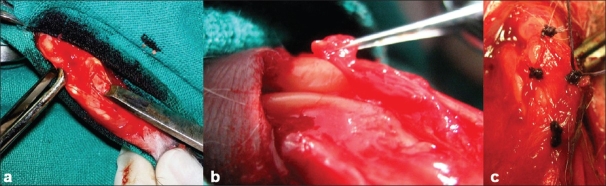
Clinical intraoperative photograph showing (a) the periosteal graft being harvested by a periosteal elevator; (b) the periosteal graft being gently slided up to the shaved articular surface of the patella; (c) the periosteal graft being sutured to the shaved articular surface of the patella

The left knee was used as the experimental side. A medial parapatellar skin incision was made starting from 1 cm above the patella and extending distally till the sufficient tibial surface was exposed to obtain the graft. After exposing, the underlying quadriceps muscle and achieving hemostasis, one medial and one lateral parapatellar incisions were made [Figure 1a]. The patella was everted laterally to 180° to expose the articular surface [Figure 1b]. Total chondrectomy on the patella up to the subchondral bone was carried out with a scalpel [Figure 1c] (complete articular cartilage of the whole of the patella was shaved till the subchondral bone). The periosteal graft was then delineated with a scalpel on the proximal third of the medial surface of the tibia. The periosteal graft was taken by stripping the periosteum gently with a periosteal elevator [Figure 2a]. Care was taken not to completely lift the periosteum off the donor site but to instead gently slide the graft up to the osteochondral defect created on the articular surface of the patella [Figure 2b]. This was necessary because once if the periosteal graft is completely lifted from the tibial surface it is not possible to identify the cambial surface of the graft with a naked eye. The graft was then sutured to the quadriceps muscle superiorly, medially, and laterally, and to the patellar tendon inferiorly [Figure 2c]. The wound was closed in layers after placing the grafted patella to its normal position.

#### Control side

The right knee joint (control) was also exposed by similar parapatellar approach and total chondrectomy up to the subchondral bone on the patella was performed after everting it to 180° and the wound was closed in layers after the reposition of the patella to its normal position.

#### Postoperative care

The animals were kept fasting for 3–4 h and were allowed to move freely in the cage. No antibiotics were given postoperatively.

The 24 experimental animals were subjected to patellectomy, 4 each at serial intervals of 2, 4, 8, 16, 32 and 48 weeks. Each specimen was coded to denote the rabbit serial number, the interval of patellectomy and the side—the control side or the grafted side.

Macroscopic observations such as the appearance of the surface, graft retention, graft adherence, and graft overgrowth were noted by two independent surgeons, who were blinded regarding the interval of patellectomy and side (control or grafted).

The specimens were then subjected to histological examination.

#### Histological technique

Each specimen was decalcified in 10% formic acid for 48–72 h and washed in running tap water for 24 h. It was then dehydrated by passing in serial ascending concentrations of 50%, 80%, and absolute alcohol for 1 h each. Xylene was used as the clearing agent. Finally, blocks were prepared in such a way so as to get saggital sections of the patella. Sections of 4–5 μm thickness were cut, using a microtome.

Slides were prepared and stained with hematoxylin and eosin stain.

Microscopic observations were noted in the form of cellularity, arrangement of chondrocytes, graft incorporation, and formation of hyaline cartilage, fibrocartilage, or fibrous tissue. The slides were observed by two independent histologists who were blinded regarding the serial interval and the side. Interobserver agreement was calculated using the Kappa test.

## RESULTS

All the rabbits survived the study. One rabbit at 4 weeks had a dislocation of the patella on the control side. None of the rabbits developed any infection or wound dehiscence.

At 2 weeks, the periosteal grafts were not adherent; however, all the grafts were *in situ*. All the grafts showed a dull surface on inspection and rough on palpation and there was no cartilage formation on histological examination. The denuded surface of control patellae in all the four rabbits appeared bare.

**Figure 3:**
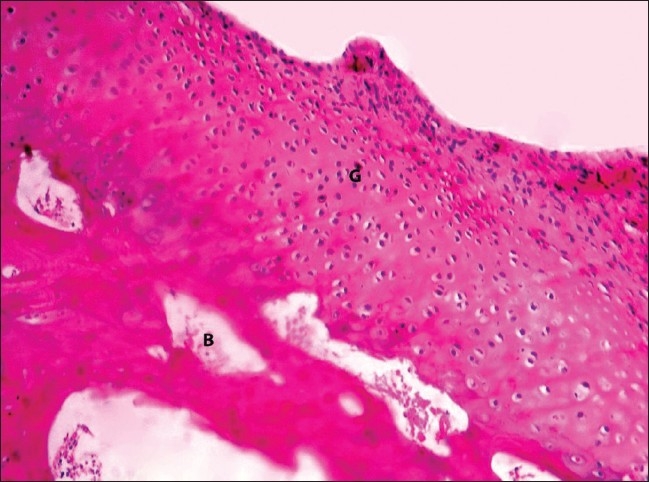
H and E-stained section (200×) of grafted patella showing the wooly arrangement of chondrocytes at 4 weeks

By the end of 4 weeks, the grafts were well incorporated macroscopically and microscopically. All the surfaces appeared glistening and smooth on palpation and there was graft overgrowth on the sides. Two grafts (50%) showed signs of hypercellularity. The cambium layer differentiated into chondrocytes and showed a wooly pattern in all the four grafts of this group [Figure 3].

In one of the rabbits, the patella of the control side got dislocated and there was fibrous tissue found macroscopically as well as microscopically on the shaved patellar surface. Another rabbit showed fibrocartilage formation on the control side.

At 8 weeks, the changes were similar to those at 4 weeks. In one rabbit, the graft uptake was absent and some fibroblasts were seen.

**Figure 4:**
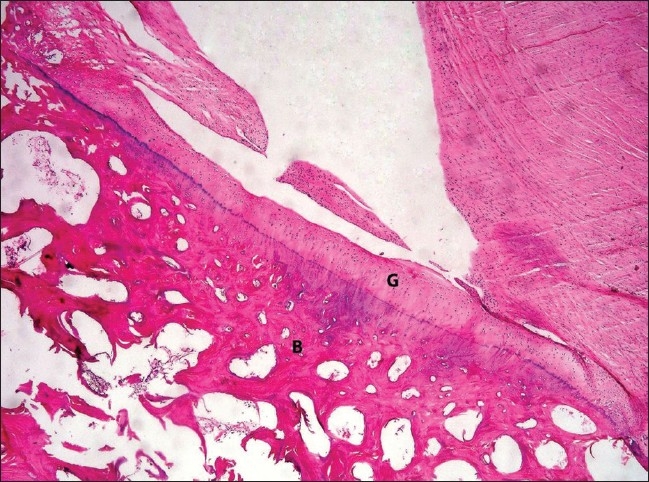
H and E-stained section (100×) of the grafted patella at 16 weeks showing chondrocytes arranging in a somewhat columnar fashion

At 16 weeks, all the grafts were adherent and well incorporated by this time. The chondrocytes were arranged in somewhat columnar fashion [Figure 4]. Two grafts (50%) were hypercellular. The other two grafts (50%) showed variability in cellularity. In one rabbit, the graft was partially displaced and showed a mixed picture of wooly and columnar patterns of cellular arrangement. The control side showed no significant changes macroscopically and microscopically.

**Figure 5:**
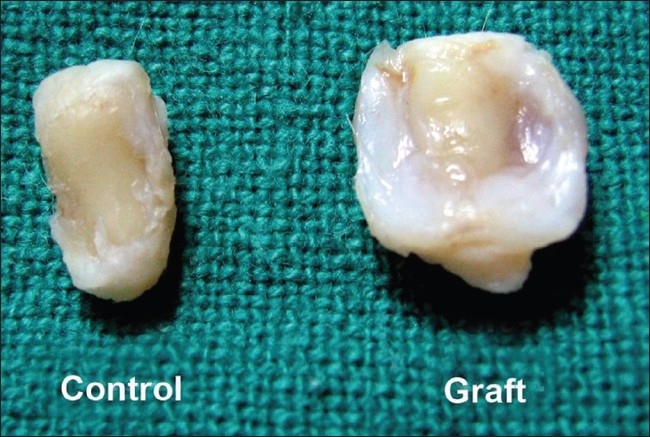
Specimen of patella showing the control and the grafted patella of rabbit no. 19 (32 weeks). The graft showed exuberant overgrowth at the periphery

At 32 weeks, all the grafts were well incorporated and three grafts (75%) showed overgrowth of the graft at the periphery [Figure 5]. Two grafts (50%) showed hypercellularity. The other two grafts (50%) showed variability in cellularity. Three grafts (75%) showed columnar orientation and one (25%) showed a wooly pattern. The control-side patellar articular surfaces remained dull and there was no formation of fibrous tissue or any type of cartilage.

**Figure 6:**
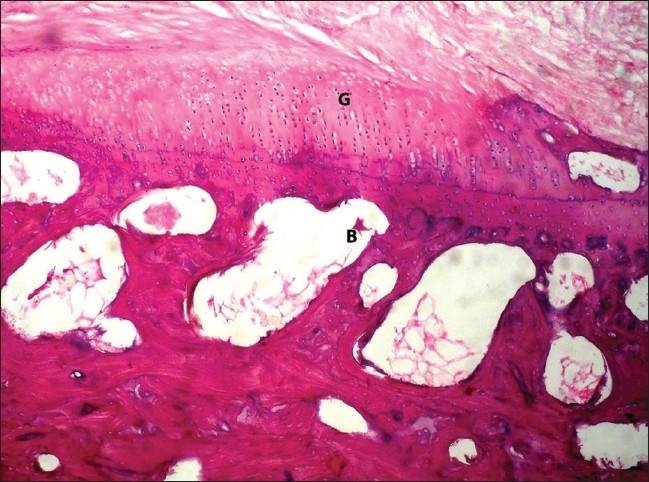
H and E-stained section (200×) of the grafted patella at 48 weeks showing hyaline articular cartilage formation which is indifferentiable from the normal articular cartilage

All the rabbits at 48 weeks showed complete adherence and excellent incorporation of the grafts. The surface appeared smooth and glistening. They had normal cellularity in three grafts (75%) and hypercellularity only in one (25%). The cells were arranged in a columnar fashion in all the grafts and assumed the microscopic picture of almost a normal articular cartilage [Figure 6]. Even at 48 weeks, the control side remained bare and did not show formation of any fibrous tissue or fibrocartilage.

The gross and histological observations are summed up in Table 1.

**Table 1:** Showing gross and histological observations

Rabbit no.	Follow-up (weeks)	Gross							Histology					Complications
	
		Graft *in situ*	Graft adherence	Graft overgrowth	Surface				Graft incorporation	Graft cellularity	Cartilage			
	
					Glistening	Dull	Smooth	Rough			Arrangement		FC/F/FB	
		
											W	CL			
1-C		–	–	–	–	+	–	+	–	–	–	–	–	–
1-G		+	–	–	–	+	–	+	–	–	–	–	–	–
2-C		–	–	–	–	+	–	+	–	–	–	–	–	–
2-G	2	+	–	–	–	+	–	+	–	–	–	–	–	–
3-C		–	–	–	–	+	–	+	–	–	–	–	–	–
3-G		+	–	–	–	+	–	+	–	–	–	–	–	–
4-C		–	–	–	–	+	–	+	–	–	–	–	–	–
4-G		+	–	–	–	+	–	+	–	–	–	–	–	–
5-C		–	–	–	–	+	–	+	–	–	–	–	–	–
5-G		+	+	–	+	–	+	–	+	Nor.	+	–	–	–
6-C		–	–	–	–	+	–	+	–	–	–	–	F	D
6-G	4	+	+	+	+	–	+	–	+	Hyp.	+	–	–	–
7-C		–	–	–	–	+	–	+	–	–	–	–	FC	–
7-G		+	+	–	+	–	+	–	+	Nor.	+	–	–	–
8-C		–	–	–	–	+	–	+	–	–	–	–	–	–
8-G		+	+	+	+	–	+	–	+	Hyp.	+	–	–	–
9-C		–	–	–	–	+	–	+	–	–	–	–	–	–
9-G		+	+	+	+	–	+	–	+	Hyp.	+	–	–	–
10-C		–	–	–	–	+	–	+	–	–	–	–	–	–
10-G	8	+	+	+	+	–	+	–	+	Hyp.	+	–	–	–
11-C		–	–	–	–	+	–	+	–	–	–	–	–	–
11-G		+	–	–	–	+	–	+	–	–	–	–	FB	–
12-C		–	–	–	–	+	–	+	–	–	–	–	–	–
12-G		+	+	+	+	–	+	–	+	Hyp.	+	–	–	–

G = Grafted side, C = Control side, AC = Articular cartilage, B = Bone, CL = Columnar, D = Dislocation, F = Fibrous tissue, FB = Fibroblastic proliferation, FC = fibrocartilage, Hyp. = Hypercellularity, Nor. = Normal cellularity, P = Patella, Var. = Variable cellularity, W = Wooly

Interobserver agreement for macroscopic and histological findings was 0.79 and 0.92, respectively.

## DISCUSSION

Cartilage regeneration still remains a challenge. Variety of methods have been described for the aforesaid, each offering specific advantages and disadvantages. These methods include autologous osteochondral grafts, osteochondral allografts, perichondral grafts, periosteal grafts, autologous chondrocyte implantation, perichondrial cells implantation, tissue engineering, undifferentiated chondrocyte precursors and gene therapy.^11^

Jaroma and Ritsila^10^ in their study concluded that free periosteal grafts on the chondrectomized articular surface of patelle differentiated into cartilage. Chondrocyte proliferation was somewhat slower in the series with the fibrous layer of the periosteum facing the subchondral bone than in the series with the cambium layer facing the articular surface. In our study, we stitched the cambium layer of the graft facing the subchondral bone believing that the mesenchymal cells will get adherent to the subchondral bone and start proliferating.

### Graft retention

In a study by Aroen *et al*.^12^ in rabbits, 16 of 23 periosteal flaps became detached within 2 weeks, with no difference in the retention rate with respect to the mobilization regime or established access to bone marrow elements in the defect. The periosteum still served as a cover of the defect in 10 of 12 knees on day 4. This figure decreased to 7 of 23 knees on day 14. However in our study, we observed that there was complete graft retention at the grafted side in all the rabbits. We believe that stitching the periosteum with the adjacent muscles gives good anchoring and retains the graft *in situ*.

### Graft adherence

Graft was adherent to the underlying bone in three rabbits (75%) at 8 weeks and four rabbits (100%) at 4, 16, 32, and 48 weeks each. Similar results were also observed by Mahadev *et al*.^13^

### Overgrowth

The graft extended beyond the patellar margins in two rabbits (50%) at 4, 16, and 48 weeks each and three rabbits (75%) at 8 and 32 weeks each. In most of the cartilage repair studies using biological implants the defects were well circumscribed. There was a congruent surface continuous with the surrounding articular surface, restored either by the graft or by host tissue overgrowth in 55.5% of the morphologically matched osteochondral allografts used in full-thickness osteochondral defects in goat knees in a study carried by Shahgaldi *et al*.^14^ Since in our study chondrectomy of the whole of the articular surface of patella and periosteal grafting with a large periosteal graft was done, there was a scope for the overgrowth of the graft beyond the patellar margins. Secondly, the patellofemoral surface pressure was not there in the marginal areas and we believe that cambium being a live tissue, once started proliferating into cartilage cells, started overgrowing.

### Surface on inspection and palpation

Three grafts (75%) at 8 weeks, and four grafts (100%) at 4, 16, 32, and 48 weeks each had a glistening and smooth surface of grafted patellae. In a study by Moran *et al*.,^15^ wherein free autogenous periosteal grafts were applied to the entire surface of patellae that had been denuded of the articular surface, the gross appearances of the articular surface of grafted patellae were identical to the normal cartilage in 87% at 6 weeks, whereas the control group did not show any signs of articular cartilage regeneration.

### Incorporation of the graft

In our study, on histological examination, the graft incorporation was seen in three rabbits (75%) at 8 weeks and four rabbits (100%) at 4, 16, 32, and 48 weeks each. In the study of chondrogenic potential of free autogenous periosteal grafts for biological resurfacing of major full-thickness defects under the influence of continuous passive motion, Driscol *et al*. observed complete bonding of the periosteal graft at 4 weeks in 80% of rabbits.^16^ It appears that in the rabbit model, a minimum period of 4 weeks is desirable for the grafts to incorporate.

### Cellularity

It appears that once the grafts became adherent by 4 weeks, they became hypercellular, maximum being by 8 weeks after which they retained a plateau and by 48 weeks they returned back to the normal cellular pattern once the articular cartilage matured.

### Arrangement of chondrocytes

Till 8 weeks the cells showed a wooly pattern of arrangement. However, from 8 weeks onward till the 48-week follow-up, it appears that there is a transition from a wooly pattern to a columnar pattern.

Three grafts (75%) at 16 and 32 weeks each showed a columnar orientation of chondrocytes and at 48 weeks, four grafts (100%) showed a beautiful columnar arrangement of chondrocytes proving that as time elapses normal cellular arrangement is achieved. Similar cellular arrangements were reported by Kumar and Chauhan in their study on the transplantation of autologous xiphisternal cartilage for the repair of articular cartilaginous defects.^17^

### Fibrocartilage/fibrous tissue/fibroblast formation

At four weeks, one rabbit (7-C) showed fibrocartilage formation. Another rabbit (6-C) showed fibrous tissue formation. This was the rabbit in which the patella got dislocated. Hence it is but natural that adhesions developed in this dislocated patella and led to the formation of the fibrous tissue. The remaining 22 control patellae did not show any type of tissue growth over the shaved patellar articular surface resembling the histological picture of osteoarthritis; these findings are similar to those of Jaroma and Ritsila^10^ and Engkvist.^18^ The patella of 11-G showed fibroblastic proliferation. In this patella the graft was not adherent.

Thus it appears that once the periosteum is transplanted in articular cartilaginous defects, the grafts are taken up by 4 weeks and the cambium layer of the periosteum starts proliferating into chondrocytes. Initially, the arrangement of the cells at 8 weeks is wooly; however, as time elapses and the graft is subjected to normal stress and strains, the cells start arranging into columnar pattern and by 48 weeks they assume the configuration of an almost normal looking hyaline articular cartilage histologically and can be a promising substitute for articular cartilaginous defects as supported by similar other studies.^19^–^21^ Methods have to be evolved to retain and fix these periosteal grafts securely intraarticularly. Considering the results of our experimental study the periosteal grafting for the repair of full-thickness articular cartilage defect may evolve into a good method.
